# Validity and reliability of the patient assessment on chronic illness care (PACIC) questionnaire: the Malay version

**DOI:** 10.1186/s12875-018-0807-5

**Published:** 2018-07-19

**Authors:** Suraya Abdul-Razak, Anis Safura Ramli, Siti Fatimah Badlishah-Sham, Jamaiyah Haniff, for the EMPOWER-PAR Investigators, Ng Chirk Jenn, Inderjit Singh Ludher, Suhazeli Abdullah, Teng Cheong Lieng, Verna K. M. Lee, Ng Kien Keat, Farnaza Ariffin, Hasidah Abdul-Hamid, Mazapuspavina Md Yasin, Nafiza Mat-Nasir, Maizatullifah Miskan, Yong-Rafidah Abdul Rahman, Mastura Ismail, Wilson H. H. Low, Sarimah Mahmood, Maryam Hannah Daud, Khamsiah Abdul Shukor, Syarifah Azimah Wan Ali

**Affiliations:** 1 Primary Care Medicine Discipline, Faculty of Medicine, Universiti Teknologi MARA, Selayang Campus, Jalan Prima Selayang 7, 68100 Batu Caves, Selangor Malaysia; 2 Institute of Pathology, Laboratory and Forensic Medicine (I-PPerForM), Universiti Teknologi MARA, Sungai Buloh Campus, Jalan Hospital, 47000 Sungai Buloh, Selangor Malaysia; 3 National Clinical Research Centre, Ministry of Health, Kuala Lumpur, Malaysia

**Keywords:** Validation, Reliability, PACIC, Cultural adaption, Malaysia

## Abstract

**Background:**

Majority of patients with chronic illnesses such as diabetes, receive care at primary care setting. Efforts have been made to restructure diabetes care in the Malaysian primary care setting in accordance with the Chronic Care Model (CCM). The Patient Assessment on Chronic Illness Care (PACIC) is a validated self-report tool to measure the extent to which patients with chronic illness receive care that aligns with the CCM. To date, no validated tool is available to evaluate healthcare delivery based on the CCM in the Malay language. Thus, the study aimed to translate the PACIC into the Malay language and validate the questionnaire among patients with diabetes in the Malaysian public primary care setting.

**Methods:**

The English version of the PACIC questionnaire is a 20-item scale measuring five key components, which are patient activation, decision support, goal setting, problem solving and follow-up care. The PACIC underwent forward - backward translation and cross cultural adaptation process to produce the PACIC-Malay version (PACIC-M). Reliability was tested using internal consistencies and test-retest reliability analyses, while construct validity was tested using the exploratory factor analysis (EFA).

**Results:**

The content of PACIC-M and the original version were conceptually equivalent. Overall, the internal consistency by Cronbach’s α was .94 and the intra-class correlation coefficient was .93. One item was deleted (item 1) when the factor loading was < 0.4. The factor analyses using promax identified three components (‘Goal Setting/Tailoring and Problem solving/Contextual’, ‘follow-up/coordination’ and ‘patient activation and delivery system design/ decision support’); explaining 61.2% of the variation. The Kaiser-Meyer-Olkin (KMO) was 0.93 and Bartlett’s test of sphericity was *p* = .000. Therefore, the final version of the PACIC-M consisted of 19 items, framed within three components.

**Conclusion:**

The findings demonstrated that the PACIC-M measured different dimensions from the English version of PACIC. It is however; highly reliable and valid to be used in assessing three CCM model subscales. Further confirmatory factor analysis of PACIC-M should be conducted to confirm this new model.

## Background

The Chronic Care Model (CCM) is being widely used to assess and improve chronic illness care in the primary care setting [1, 2]. This model represents a conceptual framework based on well documented gaps between clinical research findings and real practice [2, 3]. It recommends a proactive and planned care approach than of reactive and unplanned care, in order to deliver high quality and patient-centred chronic disease care to the population [2]. The six dimensions of CCM include healthcare organisation, delivery system design, clinical information system, patient self-management support, decision support and use of community resource [2].

The evidence on effectiveness of one or more of CCM key elements in improving Type 2 Diabetes Mellitus (T2DM) outcomes, congestive heart failure, asthma and depression are well established in developed countries [4–6]. Although the evidence of its effectiveness in developing countries are scarce, emerging evidence from Malaysia and the Philippines shows reductions of Haemoglobin A1c (HbA1C) and improvement in the proportion of patients achieving good glycaemic controls following implementation of the CCM, support restructuring of care in limited resource settings [7, 8]. With the increasing burden of chronic diseases in developing countries, measures to restructure chronic illness care using multifaceted interventions based on the CCM are required to improve delivery and quality of chronic care over time.

In Malaysia, the implementation of essential components of the CCM in the public primary care setting has been shown to be feasible [9]. The EMPOWER Participatory Action Research (EMPOWER-PAR) Study has pragmatically implemented at least three components of the CCM framework at selected public primary care clinics which include creating or strengthening the diabetes care team (delivery system design), utilizing the clinical practice guideline (CPG) by the care team (decision support) and empowering patients with self-management skills through utilization of the Global Cardiovascular Risks Self-Management Booklet© (patient self-management support) [7]. In addition, various programs by the Ministry of Health to transform the non-communicable disease care has introduced positive changes at the ground level to improve chronic care delivery including dedicated clinic specifically for T2DM [10]. There were good provisions for CPG training in most clinics, with a comprehensive national data registry for T2DM and adequacy of staff willing to be trained, providing a good opportunity for CCM implementation. With the increasing effort to restructure chronic illness care in accordance with the CCM particularly for T2DM in the Malaysian primary care setting, assessment of healthcare delivery, not only from the healthcare providers’ perspectives, but also from the patients’ perspectives are pivotal to improve chronic care quality.

The Patient Assessment Chronic Illness Care (PACIC) questionnaire is a patient reported instrument to assess quality of patient-centred care for chronic illness consistent with the CCM [11]. The PACIC is the the most appropriate instrument to measure the experience of people receiving integrated chronic care due to its psychometric characteristics, perceived applicability and relevance [12]. It was developed by Glasgow et al. in the English language and consisted of 20 items (Q1-Q20) with each item scored on a 5-point likert scale with 1 being “Almost Never” and 5 being “Almost Always”. The items were aggregated to five scale constructs that is congruent to components of the CCM but these constructs do not map perfectly onto the CCM components [11]. The five scale constructs in PACIC are patient activation; delivery system design/decision support; goal setting/tailoring; problem solving/contextual and follow-up/coordination. Each scale construct is scored by averaging the score of items answered within each scale and the overall PACIC is scored by averaging across all 20-items.

The PACIC helps healthcare providers to better understand the integration of CCM in their practices and to empower patients to be the evaluator of care they receive and avoid physicians over-reporting the CCM elements of chronic care delivered [13]. Thus, health care delivery as advocated by the CCM in the Malaysian primary care setting particularly for T2DM needs evaluation. Therefore, the objectives of this study were to translate the PACIC into the Malay language and to validate the tool among patients with T2DM receiving care at public primary care clinics.

## Methods

### Study design and participants

**Fig. 1:**
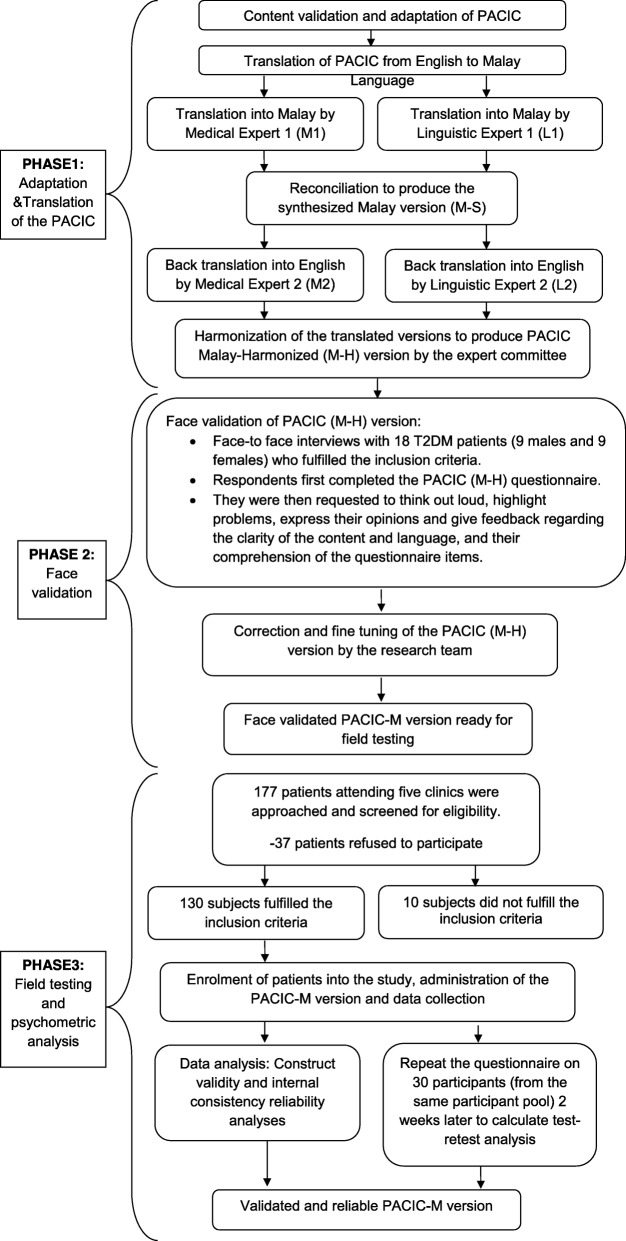
Flow chart of the conduct of the study

This cross sectional study involved three phases i) adaptation and translation ii) face validation and iii) field testing and psychometric evaluation of the PACIC-Malay (PACIC-M) version. It was conducted between March 2013 and March 2014. Figure 1 outlines the three phases of the translation and validation processes.

### Phase 1: Adaptation and translation process

The PACIC English version underwent adaptation process including content validation by a group of five family physicians. The expert panel rated the relevance of each item to the conceptual framework. Table 1 describes the rating criteria used by expert panels to assess the content validity. The rating template measures relevance, clarity, simplicity and ambiguity of PACIC-M in a scale of 1 to 4. Then, adaptations to the original questionnaire were made to suit the objectives of the study, local language and culture. The translation process into the Malay language was carried out via two forward translations. The first forward translation was done by a family physician who had direct involvement in diabetes care at public primary care clinic, and was not blinded to the study objectives. The second translator was a certified translator who was blinded to the study objectives. Each translator produced the M1 and L1 versions, which were later back translated into English independently by another certified translator and a family physician producing M2 and L2 versions, respectively. The forward and backward translations were conducted in accordance with the guidelines for cross-cultural adaptation and translation studies [14, 15]. An expert panel consisting of researchers and translators, reviewed the translated Malay versions to produce a synthesized Malay version prior to the back translation into the English language. This harmonization process ensured inter-translation validity between these versions to produce the PACIC M-harmonised version which was ready for face validation among the target population i.e. T2DM patients.

**Table 1:** Rating criteria for measuring content validity of PACIC-M

Relevance	
1 = not relevant	
2 = item need some revision	
3 = relevant but need minor revision	
4 = very relevant	
Clarity	
1 = not clear	
2 = item need some revision	
3 = clear but need minor revision	
4 = very clear	
Simplicity	
1 = not simple	
2 = item need some revision	
3 = simple but need some minor revision	
4 = very simple	
Ambiguity	
1 = doubtful	
2 = item need some revision	
3 = no doubt	
4 = meaning is clear	

### Phase 2: Face validation

The main objective of the face validation was to identify and solve any potential problems related to comprehension among the target population. The face validity testing was conducted with 18 T2DM patients (9 males and 9 females) who fulfilled the inclusion criteria. The inclusion criteria included T2DM patients aged ≥18 years who have received care at least once in the last 1 year at the public primary care clinics and were able to read and write in the Malay language. Foreigners, pregnant women and patients with Type 1 Diabetes Mellitus, mental disorders, visual impairment and those who did not give informed consent were excluded from the study. Respondents completed the PACIC M-harmonised version and they were later requested to think out loud, highlight problems, express their opinions and give feedback regarding the clarity of the content and language, and their comprehension of the questionnaire items. Following their feedback, the questionnaire was further refined to produce the final version of PACIC-M, which was ready for the psychometric evaluations.

### Phase 3: Field testing and psychometric evaluation

The final version of the PACIC-M was field tested amongst patients who fulfilled the same inclusion criteria as in Phase 2. However, patients who participated in Phase 2 and 3 were mutually exclusive, as those who participated in Phase 2 were not re-selected for Phase 3. The sampling frame for Phase 3 was T2DM patients from the five public primary care clinics participated in the EMPOWER-PAR study [7]. A detailed EMPOWER-PAR study protocol was described elsewhere [16].

### Test-retest

Thirty participants who were recruited in Phase 3 participated in the test-retest of the PACIC-M, in which they were given a date to return to the clinic after two to 4 weeks. Upon their return, they were given the same questionnaire to complete for the test-retest reliability analysis.

### Sample size

The sample size for Phase 3 was calculated using the subject to item ratio. A minimum of 100 participants were needed (20 items × 5 = 100) based on Gorsuch’s for which a subject to item ratio 5:1 was used [17]. By taking into account of a 30% non-responder rate, the sample size was increased to 130 participants.

### Sampling method

Consecutive sampling was adopted to recruit T2DM patients from the five clinics over a 2-week recruitment period i.e. 26 patients from each clinic, giving a total of 130 participants. Patients with T2DM who attended the clinic on the day of the data collection were consecutively approached and invited to participate in the study. This sampling method was chosen as there was difficulty to conduct probability sampling as the clinics only had paper based registry for their T2DM patients. A briefing to explain the purpose of the study was held to the respondents who were interested to participate and information sheets about the study were also distributed. Those who agreed to participate were then screened for eligibility according to the inclusion and exclusion criteria. Those who were eligible were recruited into the study and written informed consent was obtained.

### Data collection

Demographic data was collected and participants were given the self-administered PACIC-M questionnaire. Instructions were given on how to fill up the questionnaire and they were reminded to answer the questions themselves rather than getting their family members to complete it. Upon completion, the questionnaires were returned to the investigators who then checked for completeness.

### Statistical analysis

Data entry and statistical analysis were performed using IBM SPSS Statistics Version 21 (IBM Corp., Armonk, NY, USA). Psychometric elements of PACIC-M were then examined in three parts:

First, data was assessed for quality and data suitability for factor analysis. Data quality was assessed with mean, standard deviation, and the extent of floor and ceiling effects for all items. Floor and ceiling effects between 1 and 15% were considered as optimal [18]. Sampling adequacy was assessed using the Kaiser-Meyer-Olkin (KMO) measure value and appropriateness of data was conducted using the Bartlett’s test of sphericity. The KMO value of > 0.50 [19] with a significant Bartlett’s test of sphericity with a *p*-value of < 0.05 [20] were considered suitable for factor analysis.

Secondly, exploratory factor analysis (EFA) using principal component analysis (PCA) with promax rotation (Eigenvalue > 1) was conducted to examine the PACIC-M dimensionality and construct validity i.e. the number and type of subscales in the instrument. Only factors with values of ≥0.40 were considered. Significance of a factor loading will depend on the sample size. Typically, researchers take a loading of an absolute value > 0.3 to be important but only appropriate if the sample size is 300 [21]. With a sample size of 130, a higher loading is chosen to be considered significant. Items were excluded if they met one of the following criteria; i) weak loadings (failing to load above 0.39 on any component), ii) general loadings of 0.40 on more than one component, iii) 30% of the responses were missing or iv) 80% of item responses were the same (floor/ceiling effect).

Thirdly, reliability of the PACIC-M was assessed using internal inconsistency and test retest reliability analyses. To represent high internal consistencies, Cronbach alpha of 0.5–0.7 for groups’ comparisons [22] and average item-total correlation in a moderate range between > 0.3 and > 0.9 were considered as reliable. Cronbach alpha value of > 0.9 was considered as redundant, while correlation near 0 indicated no meaningful construct [23]. The test-retest reliability was assessed using the intra-class correlation coefficient (ICC) [24]. ICC values of > 0.7 indicated that PACIC-M was stable over time, values between 0.4–0.7 indicated fair reliability while values of < 0.4 indicated poor reliability [25]. The inter-item correlations between domains (item discriminant validity) and the overall PACIC-M (internal item convergence) was assessed using Spearman correlation due to non-normal distributions of the variables. Correlation value of ≥0.4 was considered adequate to support the internal consistency of the instrument [26].

## Results

### The content validation, translation, adaptation and face validation of PACIC-M

A group of five family physicians found that the content of all 20 items of the PACIC English version were relevant to the conceptual framework. The two forward translators from English to the Malay language agreed on most items. However, several items in the original questionnaire were adapted to suit the local language and Malaysian culture e.g. ‘healthcare team’ as used in the introductory wording is not a well-known term in the primary healthcare setting in Malaysia and among our patients. The patients might be in contact with a diabetes healthcare team but many may not be aware of this term as the team members may change regularly due to constraints in staffing faced by the public primary care clinics. The closest concept to healthcare team is healthcare providers i.e. the health clinic as a whole. Another word with ambiguous meaning was ‘treatment’ in items 1, 2, 9 and 13 in which treatment may mean medication in the day to day Malay language. The word ‘treatment’ was initially translated to ‘rawatan’, however, after the face validation process, examples of ‘treatment’ (i.e. medications, exercise, diet) were added to these items to increase the clarity. During the face validation process, all of the respondents found all of the 20 items were relevant to their chronic care. However, to improve clarity of the questionnaire, several respondents suggested that the items be changed from statements regarding their care to questions such as ‘I was asked’ to ‘Were you asked’ for all items. Following their feedback, the questionnaire was further refined and proof-read for spelling and grammar to produce the final version of PACIC-M.

### Field testing and psychometric evaluation

A total of 177 patients were approached, 37 refused to participate and 10 did not fulfil the inclusion and exclusion criteria. In total, 130 questionnaires were administered and all were returned. Table 2 shows the demographic characteristics of the respondents. The mean age was 48.5 ± 7.3 years. More than half were females (56.9%), attained secondary education (51.2%) and there were more Malays (45.7%) than other races (see Table 2).

**Table 2:** Demographic characteristics of the respondents (*N* = 130)

Characteristics	
Age in years	
Mean ± SD	48.5 ± 7.3
Median (min, max)	49.0 (32, 64)
Gender^a^ n, (%)	
Male	55 (42.6)
Female	74 (56.9)
Race^a^ n, (%)	
Malay	59 (45.7)
Chinese	21 (16.3)
Indian	47 (36.2)
Others	2 (1.6)
Educational level n, (%)	
Primary School	44 (34.1)
Secondary School	66 (51.2)
College/University	20 (14.7)
Number of co-morbid n, (%)	
0	9 (6.9)
1	46 (35.4)
2	14 (10.8)
≥ 3	61 (46.9)

^a^one missing data

Table 3 shows the descriptive statistics of the 20 item PACIC-M: mean, standard deviation (SD), percentage of respondents achieving the lowest score (indicating no satisfaction) and percentage of respondents achieving the highest score (indicating full satisfaction).

**Table 3:** Descriptive statistics of 20-item PACIC-M questionnaire, grouped into 5 subscales (*N* = 130)

Items	Mean Score	(SD)	Z-Skew	Floor N (%)	Ceiling N (%)
Overall PACIC-M score	2.53	0.48	−2.3	0 (0)	0 (0)
Patient activation	2.54	0.49	−6.4	4 (3.1)	0 (0)
Q1	2.53	0.64	−4.0	9 (6.9)	0 (0)
Q2	2.52	0.65	−4.9	11 (8.5)	0 (0)
Q3	2.55	0.62	−5.1	9 (6.9)	0 (0)
Delivery system design/decision support	2.53	0.48	−5.7	2 (1.5)	0 (0)
Q4	2.45	0.60	−2.8	7 (5.4)	0 (0)
Q5	2.48	0.64	−4.0	10 (7.7)	0 (0)
Q6	2.64	0.57	−6.2	6 (4.6)	0 (0)
Goal setting/tailoring	2.53	0.46	−4.6	0 (0)	0 (0)
Q7	2.52	0.60	−4.0	7 (5.4)	0 (0)
Q8	2.56	0.60	−4.8	7 (5.4)	0 (0)
Q9	2.46	0.66	−4.0	12 (9.2)	0 (0)
Q10	2.55	0.61	−4.7	8 (6.2)	0 (0)
Q11	2.55	0.61	−4.7	8 (6.2)	0 (0)
Problem solving/contextual	2.52	0.51	−4.3	0 (0)	0 (0)
Q12	2.48	0.61	−3.4	8 (6.2)	0 (0)
Q13	2.52	0.60	−3.9	7 (5.4)	0 (0)
Q14	2.54	0.64	−5.0	10 (7.7)	0 (0)
Q15	2.54	0.68	−2.8	11 (8.5)	1 (0.8)
Follow-up/coordination	2.49	0.60	−5.2	6 (4.6)	0 (0)
Q16	2.40	0.75	−3.8	21 (16.2)	0 (0)
Q17	2.51	0.67	−4.9	13 (10.0)	0 (0)
Q18	2.58	0.71	−6.0	16 (12.3)	0 (0)
Q19	2.48	0.70	−3.9	14 (10.8)	0 (0)
Q20	2.46	0.71	−4.4	16 (12.3)	0 (0)

There was no missing data for individual items for PACIC-M. The floor effect (percent of patients answering “None of the time” to any given item) exceeded 15% in item 16 and the ceiling effect (percent of patient answering “All of the time” to any given item) was not prominent, with none of the item exceeded 15%.

Overall, PACIC-M score was 2.53 (±0.48) out of possible score of 5, ranging between 2.45 (±0.60) for item 4: “Given a written list of things I should do to improve my health” and 2.64 (±0.57) for item 6 “Shown how what I did to take care of myself influenced my condition”. When grouped together as five predetermined subscales, the subscale score ranged between 2.49 (±0.60) for subscale 5: “follow-up/coordination” and 2.54 (±0.49) for subscale 1: “patient activation”. Floor and ceiling effect for the entire PACIC-M was 0% respectively.

The KMO revealed an excellent value of 0.93 and the Bartlett’s test of sphericity value was significant (1565.7, *p* = .000). Both of these values indicated that the data set was suitable for further factor analysis. Using the PCA with promax rotation, we identified three components explaining 61.2% of the total variance. The first component with Eigenvalue of 9.853 explained 49.3% of the total variance, while the second component with an Eigenvalue of 1.379 explained 6.9% of the total variance. The third component with Eigenvalue of 1.012 explained 5.1% of the total variance. Table 4 shows the results of factor loadings of the PACIC-M 3-component structure. Factor loading for item 1 was below 0.4 for all components identified de-novo. Component one which consisted of items 6,7,8,9,10,11,12,13,14 and 15 was labelled as ‘goal setting/tailoring and problem solving/contextual’. Component two which consisted of items 16, 17, 18, 19 and 20 was labelled as ‘follow-up/coordination’. The third component which consisted of items 2, 3, 4 and 5 was labelled as ‘patient activation and delivery system design/ decision support’.

**Table 4:** Factor loadings of the PACIC-M reveals 3-component structure

	Component		
	1	2	3
Q1	0.307	0.218	0.215
Q2	−0.255	0.063	**0.915**
Q3	0.138	−0.101	**0.783**
Q4	0.055	0.009	**0.659**
Q5	0.329	0.006	**0.546**
Q6	**0.410**	0.336	0.198
Q7	**0.606**	0.001	0.069
Q8	**0.546**	0.328	−0.147
Q9	**0.901**	−0.225	0.053
Q10	**0.576**	0.210	−0.006
Q11	**0.573**	0.202	−0.014
Q12	**0.745**	0.071	−0.071
Q13	**0.722**	0.108	−0.073
Q14	**0.515**	0.295	0.030
Q15	**0.870**	−0.110	−0.006
Q16	0.295	**0.465**	0.015
Q17	−0.161	**0.906**	0.150
Q18	−0.023	**0.833**	0.109
Q19	0.037	**0.938**	−0.155
Q20	0.041	**0.908**	−0.062

Extraction Method: Principal Component Analysis

Rotation Method: Promax with Kaiser Normalization

Note: Factor loadings > 0.40 appear in bold

Table 5 shows the score distributions and reliability of PACIC-M. The Cronbach alpha value for the overall PACIC-M was 0.94 and ICC was 0.93. The Cronbach alpha values for two subscales ‘goal setting/tailoring and problem solving/contextual’ and ‘follow-up/coordination’ was greater than 0.9, respectively which is high for a brief scale [22]. One subscale i.e. ‘patient activation and delivery system design/decision support’ had a Cronbach alpha value of 0.77.

**Table 5:** Reliability of PACIC-M

	Cronbach alpha	Intra-class correlation
Overall PACIC-M	0.94	0.93
PACIC-M Scales		
Goal setting/tailoring and problem solving/contextual	0.91	
Follow-up/coordination	0.90	
Patient activation and delivery system design/ decision support	0.77	

Table 6 shows the Spearman correlations between the original PACIC subscales and the overall score. The correlations between the overall and PACIC subscales were found to be generally higher than correlations between subscales. Correlations between subscales were positive but lower, ranging between 0.41 for ‘patient activation’ and ‘problem solving/contextual’, and 0.80 for ‘goal setting/tailoring’ and ‘problem solving/contextual’.

**Table 6:** Spearman correlations between the original PACIC subscales and the overall score

	Patient activation	Delivery system design/decision support	Goal setting/tailoring	Problem solving/contextual	Follow-up/coordination
Patient activation	1	.623^a^	.455^a^	.408^a^	.523^a^
Delivery system design/decision support		1	.587^a^	.582^a^	.589^a^
Goal setting/tailoring			1	.807^a^	.689^a^
Problem solving/Contextual				1	.692^a^
Follow-up/coordination					1
Overall score	.671^a^	.748^a^	.877^a^	.858^a^	.872^a^

^a^Correlation is significant at the 0.01 level (2-tailed)

**Fig. 2:**
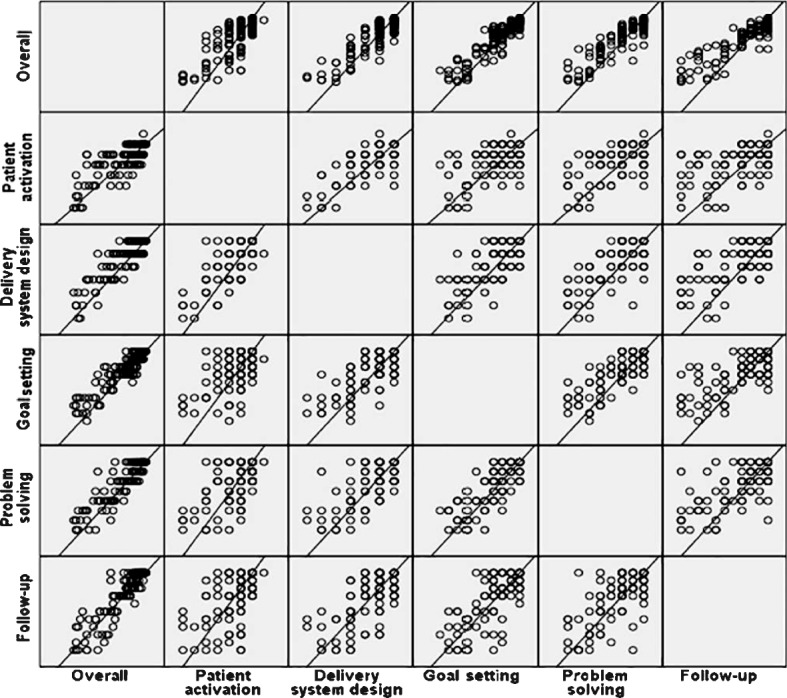
Matrix plot of mean score for overall, patient activation, decision support, goal setting and follow up

Meanwhile, Fig. 2 shows the summary of the matrix scatter plot of mean score for PACIC subscales.

## Discussion

The implementation of CCM to guide chronic disease management system change for T2DM in the Malaysian primary care setting highlights a pressing need for a practical and validated tool to evaluate the quality of patient-centred care which is consistent with the CCM. This was the first study to translate, adapt and validate the PACIC questionnaire into the Malay language among patients with T2DM whom received care at the public primary care clinics in Malaysia. The PACIC-M had undergone rigorous process according to established guidelines for translating, adapting and validating a questionnaire [14, 15].

Our study shows that the overall and individual subscales PACIC-M scores were comparable to other studies [11, 27]. The floor and ceiling effect in this study was better than studies conducted in the Danish populations [28, 29], and similar to a Spanish validation of PACIC delivered in ambulatory clinic where the staff received intensive training in CCM [30]. Our findings have also shown that the internal consistency for overall PACIC-M is very high and comparable to the original PACIC instrument and to most of the other validated PACIC done in various European countries [11, 27, 29]. The instrument is stable over time which is similar with Glasgow et al. [11] and Rosemann et al. [27] The inter-item correlations among the subscales were found to be high and this is comparable to other studies [11, 27].

The final validated PACIC-M consisted of 19 items framed within the following three components i) goal setting/tailoring and problem solving/contextual, ii) follow-up/coordination, and iii) patient activation and delivery system design/ decision support. Item 1 was removed as it did not load onto any of the three components. Like others who translated the PACIC into different languages [31–33], we were unable to confirm the 5-component structure model in the factorial examination in patient responses to PACIC-M instrument. PACIC-M was unable to identify all components of the CCM, in which it was inadequate to provide information about individual construct of CCM as expected. Our findings suggest almost half of the PACIC-M variability was explained by component 1 (item 6 to15), which is a combination of ‘goal setting/tailoring and problem solving/contextual’. This component is positively correlated to component 2 (follow-up/coordination) and component 3 (patient activation and delivery system design/decision support). Our findings of the 3-component structure of the PACIC-M is a reflection of the EMPOWER-PAR intervention which reinforced the national clinical guideline recommendations for diabetes care through individualized goal setting of treatment targets, problem solving skills, follow-up and coordination of care by the healthcare team [7, 16]. It also reflects the use of Global Cardiovascular Risks Self-Management Booklet which supports goal setting and problem solving skills through effective communication and patient empowerment [7, 16]. These three components were found to be positively correlated which signifies prominence of these three subscales in the selected clinics.

In the development of PACIC, Glasgow et al. conducted confirmatory factor analysis (CFA) and concluded that the five scales identified were moderately fit [11]. It was further highlighted that the correlation and reliability coefficients reported by Glasgow et al. underestimated the true parameters when Cronbach’s alpha and Pearson’s correlation was used when a normal data from ordinal scale was assumed [34]. It was argued that the 5-point likert score used to measure the score was ordinal but was used in the manner appropriate for interval measurement, in which interpretation of mean score used by Glasgow et al. should be based upon equidistance between two points [34, 35]. Subsequently, published factor analyses found conflicting results, in which only two out of seven studies reported unequivocal results to support the 5-factor structure while others suggested a variety of techniques for further validation [35]. It was subsequently translated and validated in the primary care setting among Danish populations [28, 29, 31] and German populations [27]. These studies have shown mixed results and further validation study was suggested.

Our study highlights an interesting finding when patients’ responses may not conform to the expectations of the design of the questionnaire, such that analyses of the responses can reveal aspects of care delivery deemed important for the patients. Patients may be conscious of clinical targets and self-care as dominated by the two subscales following utilization of the self-management booklet but may be unaware of system delivery re-design which is important to healthcare providers. The 20-items of PACIC was designed to assess patients’ perspectives, of which patient’s experiences of chronic illness care and their understanding of CCM concept is substantial in order to match the person interpreting the results. This finding affirms that patients understanding and interpretation may vary and may be influenced by individual patient’s factor and care delivery already implemented in the chronic disease management system. However, confirmatory factor analysis of the PACIC-M is recommended to confirm the 3-component model found in this study.

### Study limitations

This study has several limitations. The final PACIC-M consisted of 19 items, with Item 1 was deleted when the factor loading was < 0.4. We consider the deletion is appropriate when significance of a factor loading will depend on the sample size, in which a higher loading is needed if the sample size is small [21]. Typically, researchers take a loading of an absolute value > 0.3 to be important, and only appropriate if the sample size is 300 [21]. With a sample size of 130, a higher loading was chosen to be significant for our study. Secondly, the PACIC-M was administered to T2DM patients who were able to read and understand the Malay language. Therefore, the findings of this study could only be generalised and the usability of PACIC-M could only be extended to individuals with T2DM who could read and understand the Malay language. There is a need to translate and validate this questionnaire into other languages such as Mandarin and Tamil to give better utilisation in a multi ethnic Malaysian population. For item discriminant validity, we were not able to test the hypothesized scales with other measurement tool as none were available in the Malay language. Other limitation includes the consecutive sampling method used in this study which may be vulnerable to sampling bias. However, measures were taken to ensure that all T2DM patients who attended the clinics on the data collection days were approached and invited to participate.

### Implications for clinical practice and future research

The validated PACIC-M serves as an important patient reported instrument to measure the quality of patient-centred care for T2DM which is consistent with the CCM in the Malaysian primary care setting. This information would be pivotal in guiding the healthcare professionals and policy makers to make the necessary changes to the chronic disease delivery system, to ensure that patients are satisfied with the care that they receive. However, to strengthen the validity of the PACIC-M, further validation study which includes confirmatory factor analysis is recommended. Future research may also include utilisation of the PACIC-M to evaluate the impact of a CCM-based intervention on the perceived quality of care as received by the patients.

## Conclusions

The PACIC-M contains 19 items which are framed within 3-component model. It is a valid and reliable tool which can be used to measure the perception of T2DM patients towards the care that they receive and whether the care is congruent with the CCM elements. However, further validation study which includes confirmatory factor analysis is recommended to strengthen the validity of the PACIC-M.

## Abbreviations

CCM: Chronic care model
CFA: Confirmatory factor analysis
CPG: Clinical practice guideline
EFA: Exploratory factor analysis
EMPOWER-PAR: EMPOWER participatory action research
HbA1C: Haemoglobin A1c
ICC: Intra-class correlation coefficient
KMO: Kaiser-Meyer-Olkin
MOH: Ministry of Health
PACIC: Patient assessment on chronic illness care
PACIC-M: PACIC-Malay version
PCA: Principal component analysis
Q1-Q20: Item 1-item 20
SD: Standard deviation
T2DM: Type 2 diabetes mellitus
UiTM: Universiti Teknologi MARA
